# Malignant teratoma in Klippel–Feil syndrome: a case report and review of the literature

**DOI:** 10.1186/s13256-015-0700-y

**Published:** 2015-10-04

**Authors:** A. Adorno, C. Alafaci, F. Sanfilippo, D. Cafarella, M. Scordino, F. Granata, G. Grasso, F M Salpietro

**Affiliations:** 1 Department of Neurosurgery - AOOR Papardo-Piemonte, Contrada Papardo, 98158 Messina, Italy; 2 Department of Neurosurgery - AOU Policlinico “G.Martino”, Via Consolare Valeria, 98125 Messina, Italy; 3 Department of Neuroradiology, University of Messina, Messina, Italy; 4 Department of Neurosurgery - BIONEC, University of Palermo, Palermo, Italy

**Keywords:** Cervical spine, Dermoid tumor, Klippel–Feil syndrome, Pathology, Posterior fossa, Teratoma

## Abstract

**Introduction:**

Klippel–Feil syndrome is characterized by a congenital fusion of cervical vertebrae. Intracranial teratomas are nongerminomatous germ cell tumors and they account for 0.3 to 0.9% of all intracranial tumors. Teratomas with malignant transformation refer to lesions which give rise to malignant cancer of somatic type. The association between tumors of dermoid origin and Klippel–Feil malformation is extremely rare. Only 23 other cases have so far been reported, and only one case of dermoid tumor with areas of dedifferentiation on squamous cell carcinoma has been described.

**Case presentation:**

We report the case of a 72-year-old white man with a 2-year history of gait and balance disturbances. A brain magnetic resonance imaging revealed a fourth ventricle neoplastic process with infiltrative features. He was operated through a suboccipital craniectomy with a C1 laminotomy and bilateral vertebral artery transposition. At 6-months follow-up, magnetic resonance imaging showed an early regrowth of the fourth ventricle tumor, with the same radiological features.

**Conclusions:**

Patients with Klippel–Feil malformation could develop posterior fossa dermoid tumors. The malignant potential of such tumors must be considered and surgery is recommended. Particular attention must be focused on the histopathological analysis in order to identify possible foci of malignant transformation.

## Introduction

Klippel–Feil syndrome is characterized by a congenital fusion of cervical vertebrae; it was described from autopsy findings in 1912. It is clinically characterized by the classic triad of brevicollis, low posterior hairline and severe restriction of neck motion in combination with two or more non-segmented cervical vertebrae [1].

Intracranial teratomas account for 0.3 to 0.9% of all intracranial tumors [2]. They are nongerminomatous germ cell tumors that contain areas corresponding to endodermal, mesodermal and ectodermal differentiation. Mature teratomas are benign tumors with weak or absent mitotic activity, composed of fully differentiated tissues derived from all three germ layers. Although immature teratomas are known to constitute 10 to 50% of teratomas, those arising from the central nervous system (CNS) are exceedingly rare. Thus only few case reports or limited clinical series have dealt with these uncommon neoplasms [3, 4]. They are characterized by the presence of incomplete differentiated, embryonic or fetal tissues. Teratomas with malignant transformation refer to lesions that give rise to a malignant tumor of somatic type, most commonly a rhabdomyosarcoma, a squamous cell carcinoma or an adenocarcinoma. Intracranial teratomas mimic other intracranial germ cell tumors in their proclivity for the midline, most often arising in the pineal gland and suprasellar region and, less frequently, followed by third and fourth ventricle [4].

The first association between posterior fossa dermoid tumors and cervical fusion anomaly was described by Love and Kernohan [5]. This association is extremely rare and, since then, only 23 cases have been reported [5–14]. Only one case of dermoid tumor with an area of differentiation in squamous cell carcinoma has been reported [15].

## Case presentation

A 72-year-old white man presented with a 2-year history of gait and balance disturbance, associated with paresthesia to his upper and lower limbs and frequent episodes of falling to the ground. He recently referred episodes of morning vomiting. At a physical examination he presented with a short neck and a low hairline at the back of his head. A neurological examination revealed ataxic gait with retropulsion at the Romberg test, slight weakness in his right limbs, tetra hyperreflexia with bilateral Hoffmann and Babinski signs, and dysmetria in the finger-to-nose test. A magnetic resonance imaging (MRI) study revealed a fourth ventricle neoplastic lesion, with infiltrative features and non homogeneous signal. The lesion presented a uniform contrast enhancement in its posterior part while a peripheral enhancement was observed in its anterior portion.

**Fig. 1:**
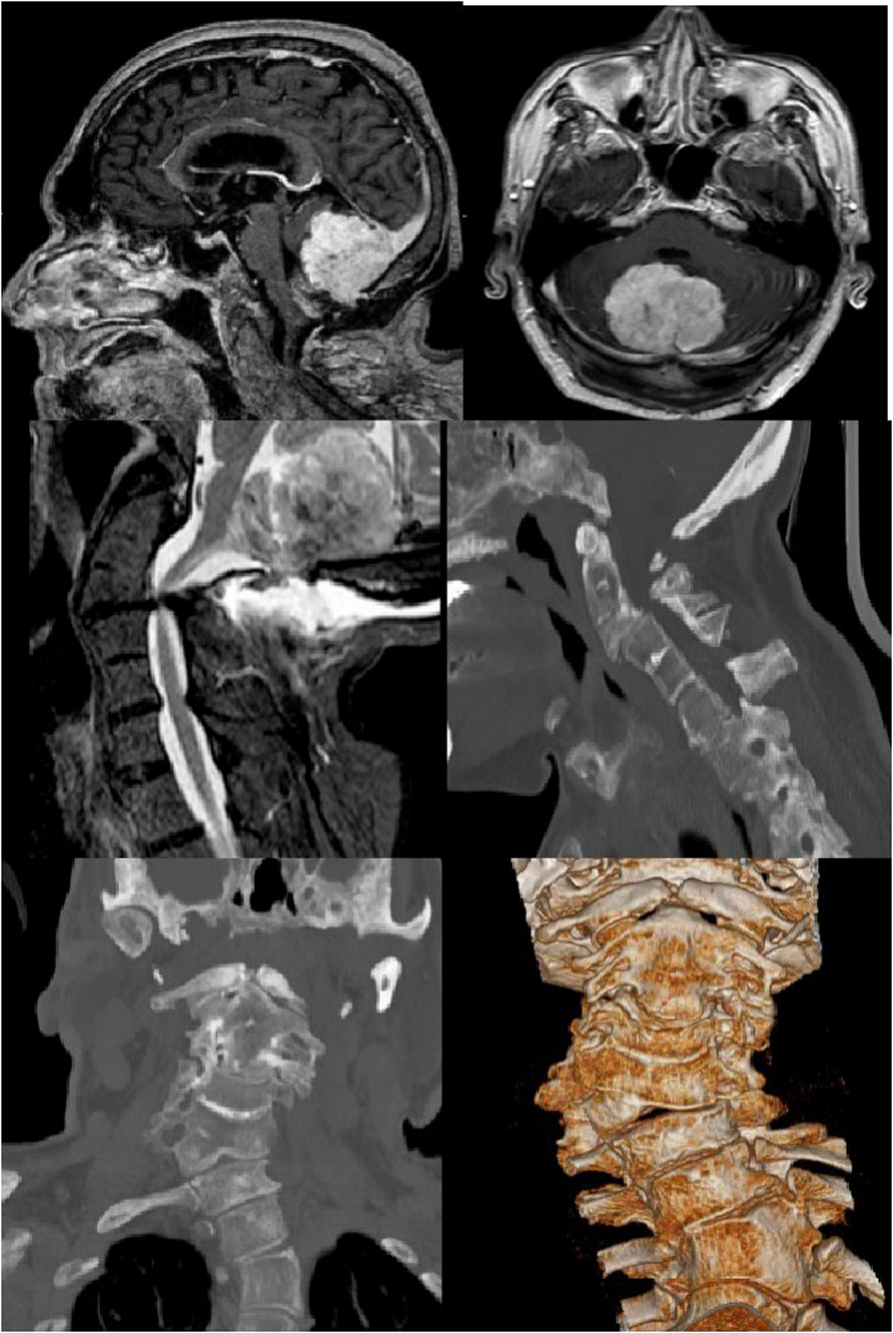
Sagittal (*upper left)* and axial (*upper right*) contrast-enhanced T1-weighted magnetic resonance imaging showing a fourth ventricle tumor with infiltrative features and non homogeneous signal. Sagittal craniocervical T2-weighted magnetic resonance imaging *(middle left*), sagittal craniocervical scanning (*middle right*) and coronal computed tomography images (*lower right and left*) revealing a scoliotic deformation of the vertebral column with C3 to C4 synostosis

A craniocervical computed tomography (CT) scanning revealed a scoliotic deformation of the vertebral column with C3 to C4 synostosis. These features allowed a diagnosis of Klippel–Feil syndrome (Fig. 1).

The patient was operated through a suboccipital craniectomy with a C1 laminotomy and bilateral vertebral artery transposition. We performed a subtotal tumor resection using a telovelar subtonsillar route, because of tight neoplastic adhesions to the cranial portion of the floor of the fourth ventricle.

**Fig. 2:**
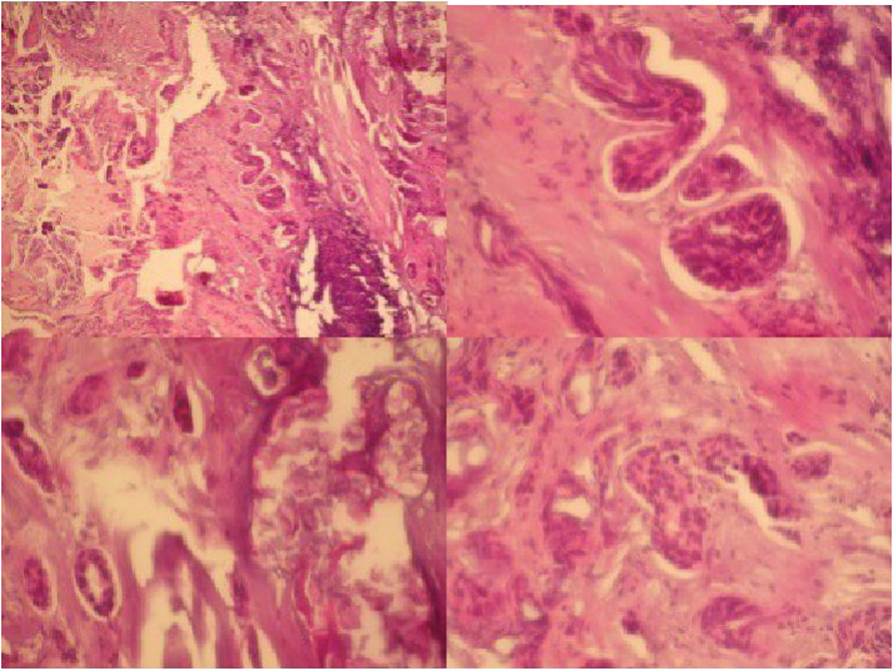
Images showing a periodic acid–Schiff-positive dysmorphic material with pluristratified epithelial islands. A teratoma, partly cystic, with pseudo cartilage areas and hair and tooth-like formations was identified. There were also features of a squamous cell carcinoma with atypical polymorphic epithelial cells, necrosis and focal cornification

A histopathological examination revealed a periodic acid–Schiff (PAS)-positive dysmorphic material with pluristratified epithelial islands. A teratoma, partly cystic, with pseudo-cartilage areas and hair and tooth-like formations was identified. There were also features of a squamous cell carcinoma with atypical polymorphic epithelial cells, necrosis and focal cornification. The lesion was related to a teratoma with malignant transformation (Fig. 2).

**Fig. 3:**
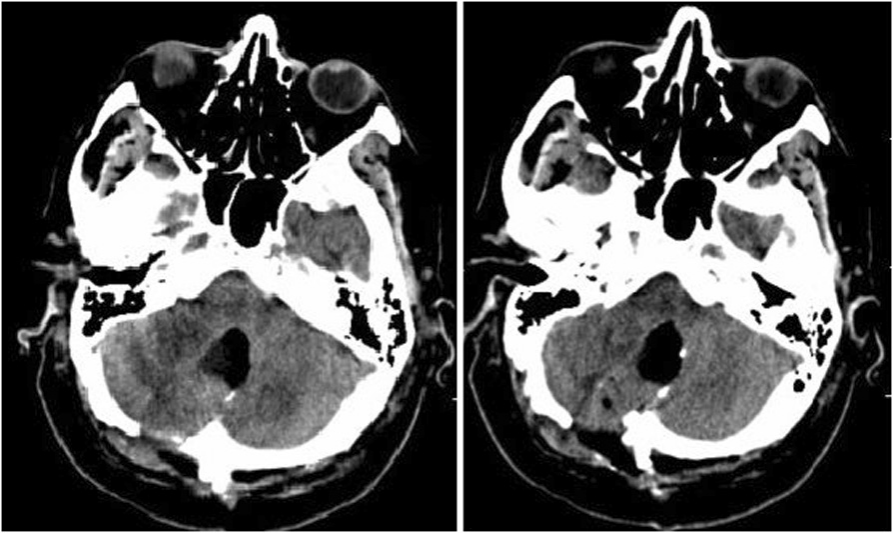
Postoperative computed tomography image demonstrating a partial removal of the lesion

The postoperative course was uneventful with neurological improvement. The patient was able to walk without ataxic feature and the Romberg test was improved. A postsurgical CT scan demonstrated a partial removal (Fig. 3).

**Fig. 4:**
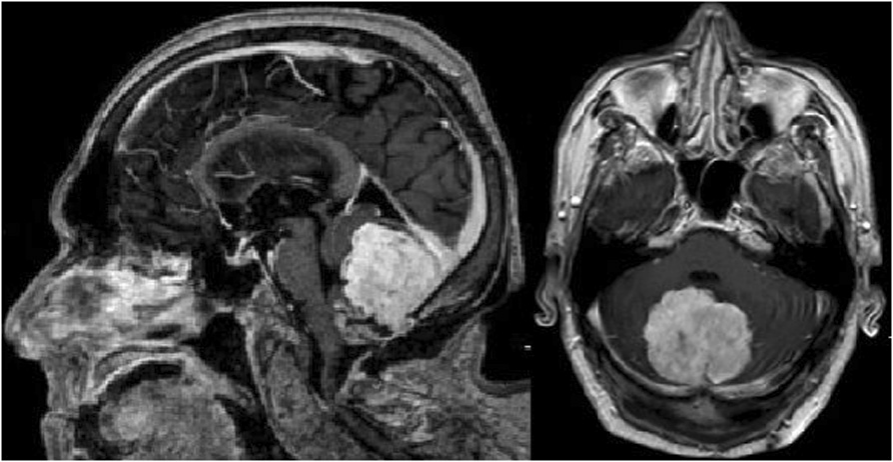
At 6-months follow-up, a magnetic resonance imaging showed an early regrowth of the fourth ventricle tumor

At 6-months follow-up, a MRI showed an early regrowth of the fourth ventricle tumor, with the same radiological features (Fig. 4). According to the wishes of the patient and his relatives, no further surgical treatment or adjuvant therapy was performed.

## Discussion

The association between posterior fossa dermoid tumor and Klippel–Feil syndrome is well known. However, the association of cervical fusion anomalies with malignant teratomatous lesions has rarely been reported.

Many hypotheses have been proposed to explain the embryological association of the two pathological conditions. One theory suggests that failure of the segmentation of the cervical sclerotomes could lead to the Klippel–Feil anomaly. In accordance with this, the absence of the cleavage of the ectoderm from the neuroectoderm would result in entrapment of the dermal elements within the closing neurotube [11]. Another theory suggests that during the formation of the cephalic and cervical brain flexures, a shortening of the cervical spine related to the fusion of the somites, may result in altered tissue tension. This process would lead to the entrapment of immature dermal elements [16]. Following the neural tube overdistension and somites distortion, the expression of *Hox* and *Pax* genes, the DNA sequences that control the development of intervertebral disk may be reduced producing the vertebral segmentation. It has also been speculated that this occurrence, during the 28th to 35th days of life, may cause an altered tissue tension at the cervicomedullary junction resulting in the entrapment of dermal elements. This process may be responsible for the formation of posterior fossa dermoid tumors [10, 16].

There are different mechanisms underlying the migration of germ cells including pseudopodial structures, chemotactic factors, basement membrane or extracellular molecules [16].

Another important structure involved in posterior fossa embryological development is the isthmus; the isthmus develops at the junction of the mesencephalon and metencephalon, thus serving as an organizing center for both midbrain and hindbrain differentiation. The homeobox genes play a crucial role in segmentation and subsequent patterning. Among them, the Sonic hedgehog regulates the midbrain–hindbrain morphogenesis through positive regulation of the Gli activators (GLI1) and inhibition of the Gli repressors (GLI3) and controls the overall growth of this region. Sonic hedgehog also restricts FGF8 expression to the isthmus, which is essential for the differentiation of the tecto-isthmo-cerebellar region [17]. The encoded protein SNF5 has an oncosuppressor role by cooperating with p53 in the inhibition of GLI1; the loss of SNF5 causes the aberrant activation of the Sonic hedgehog pathway and drives teratoid tumorigenesis through the expression of GLI1, as documented by the growth of SNF5-deficient malignant rhabdoid cells *in vivo* and *in vitro* [18]. Sonic hedgehog and GLI1 are also crucially involved in the morphogenesis of the tecto-cerebellar midline structures, as previously mentioned. The correlation between embryogenesis and tumorigenesis is well characterized by the involvement of Sonic hedgehog signaling in medulloblastoma [18].

In our case we report an early tumor regrowth, after a subtotal removal, due to tight adherences of the tumor to the floor of the fourth ventricle. The extremely aggressive features of this kind of lesion have to be related to the nature of teratomatous tumors. The so-called “growing teratoma syndrome” is a rare complication which follows the resection of a recurrent intracranial nongerminomatous germ cell tumor in adults [19]. It is observed after partial response to multimodality therapy and despite a decrease in tumor serum markers. In these cases, the enlarging tumors consist of elements of mature teratoma that presumably are refractory to chemotherapy or radiation, and are selected by these same modalities of treatment. In our case, the tumor regrowth was caused by the surgical treatment, due to the removal of the less aggressive intraventricular tumor leaving the most aggressive portions of the tumor, which proliferate very quickly.

## Conclusions

The early regrowth of the tumor from a small postsurgical remnant demonstrates that inside a posterior fossa teratoid lesion, there are some more aggressive areas with malignant transformation. Complete removal of these islands adherent to the floor of the fourth ventricle is extremely dangerous and a total removal is very difficult. The association of posterior fossa teratoid lesion with Klippel–Feil disease is very rare and represents a surgical challenge.

## Consent

Written informed consent was obtained from the patient for publication of this case report and any accompanying images. A copy of the written consent is available for review by the Editor-in-Chief of this journal.
